# Construction of tissue-engineered laryngeal cartilage with a hollow, semi-flared shape using poly(3-hydroxybutyrate-co-3-hydroxyhexanoate) as a scaffold

**DOI:** 10.3892/etm.2015.2262

**Published:** 2015-02-05

**Authors:** ANKE SUN, QINGYAN MENG, WANTONG LI, SONGBO LIU, WEI CHEN

**Affiliations:** 1 Department of Otolaryngology, General Hospital of Shenyang Military Area Command, Shenyang, Liaoning 110016, P.R. China; 2 Department of Burns and Aesthetic Medicine, General Hospital of Shenyang Military Area Command, Shenyang, Liaoning 110016, P.R. China; 3 Department of Microsurgery, General Hospital of Shenyang Military Area Command, Shenyang, Liaoning 110016, P.R. China; 4 Department of Experimental Medicine, General Hospital of Shenyang Military Area Command, Shenyang, Liaoning 110016, P.R. China

**Keywords:** poly (3-hydroxybutyrate-co-3-hydroxyhexanoate), artificial, laryngeal cartilage, myofascial flap, semi-flared shape, tissue engineering

## Abstract

The aim of the present study was to construct tissue-engineered laryngeal cartilage with a hollow, semi-flared shape using a poly(3-hydroxybutyrate-co-3-hydroxyhexanoate) (PHBHH) scaffold. Porous PHBHH was prepared and formed into a hollow, semi-flared shape, and the cell-material composites were cultured for one week *in vitro* prior to implantation *in vivo*. Cells of the nine rabbits of the experimental group were filled and encapsulated in the myofascial flap-shaping material composite for *in situ* implantation. The three rabbits in the control group were treated with the shaping material without the chondrocytes. Cartilage regeneration was assessed at six, 12 and 18 weeks after surgery. In the experimental group, the shape and porosity of the material were ideal, the cells exhibited good adhesion with the material and the myofascial flap blood supply was rich. The engineered laryngeal cartilage with the hollow, semi-flared shape was ideally formed, and the cartilage formed at six weeks after the surgery. Further maturation of the cartilage was observed at 12 and 18 weeks after the surgery. PHBHH was a suitable material for the formation of a hollow, semi-flared shape with good cellular compatibility. Myofascial flap filling and wrapping can be used to build tissue-engineered laryngeal cartilage with a hollow, semi-flared shape.

## Introduction

The study of tissue-engineered cartilage with the basic goals of predetermined shaping and regeneration has provided novel ideas and techniques for research into laryngeal cartilage erosion (1–3); however, due to the special nature of the morphology, location and function of laryngeal cartilage, tissue engineering research has not, to date, exhibited its full advantages in the reconstruction of laryngeal cartilage (4). This is not only due to the large quantity of seed cells and biomaterials required for the construction and shaping of the tissue-engineered laryngeal cartilage, but also due to the difficulty of constructing the ideal irregular, hollow, three-dimensional structure. Tissue-engineered laryngeal cartilage with a hollow, semi-flared shape should show good biocompatibility, biodegradability and structures of the pores inside the biological materials, as well as sufficient mechanical strength and flexibility (5). How to ensure that the cartilage maintains its shape and structure and how to develop the potential of cartilage reshaping applications are two problems currently faced in the process of tissue-engineered, hollow, semi-flared laryngeal cartilage construction. The exploration of hollow structural biomaterials that are more suitable for shaping and tissue-engineered cartilage regeneration, as well as the practical application of the cartilage tissue engineering regenerative technology, is the only way to improve laryngeal cartilage repair and reconstruction. In the present study, on the basis of previously published tissue engineering findings (6,7), the new biomaterial poly(3-hydroxybutyrate-co-3-hydroxyhexanoate) (PHBHH) was used in the shaping of laryngeal tissue-engineered cartilage tissue into a hollow, half-flared shape, and the compatibility of the PHBHH with chondrocytes was investigated. The aim of the study was to determine the feasibility of constructing laryngeal cartilage tissue with a hollow, half-flared shape using the muscle wrapping and filling method and using the fascia lining of a pedicled myofascial flap, in order to provide an experimental basis for the tissue engineering of laryngeal cartilage with a hollow, half-flared shape through the transplantation of myofascial tissue.

## Materials and methods

### Chondrocyte harvest

Costal and articular cartilages were obtained under sterile conditions from young New Zealand rabbits of both genders, aged between three and seven days, provided by the Center of Laboratory Animals, Shenyang Military Area Command (Shenyang, China). The cartilage was cleaned, washed twice with 0.1 mol/l sterile phosphate-buffered saline (PBS) containing 200 U/ml penicillin and streptomycin, respectively, and digested with 0.25% trypsin (Sigma-Aldrich, St. Louis, MO, USA) for 2 min. Following digestion, the cartilage was washed with PBS a further three times, cut into pieces (1–2 mm^3^) and washed again with PBS. The pieces were then placed in a small 50-ml beaker. Type II collagenase (0.3%; Sigma-Aldrich) was added and stirred for digestion at 37°C, and the cells were subsequently collected once every 0.5 h, starting from 1 h, until all the solids had disappeared. The cell suspension was obtained and centrifuged at 250 xg for 10 min, the supernatant was discarded and the precipitate was washed with PBS two times. Dulbecco’s Modified Eagle’s medium/Ham-F12 (1:1) mixed culture medium (Gibco-BRL; Invitrogen Life Technologies, Carlsbad, CA, USA), containing 20% fetal bovine serum (Zhejiang Evergreen Biologicals, Hangzhou, China), was used to resuspend the cells, and trypan blue staining was performed. The cells were counted using a cell counting board; stained cells were considered to be non-viable cells while unstained cells were considered to be viable). Cells were seeded in 100-ml culture flasks at a density of 2×10^5^ cells/ml and cultured in a humidified CO_2_ incubator (Thermo Fisher Scientific Inc., Waltham, MA, USA). The medium was changed every 48 h, and the cells were passaged when the whole wall of the culture flask was covered by adhered cells.

### Mold preparation

Shaping was performed in reference to the full morphology of adult laryngeal cartilage, and the plastic models were prepared by 3:1 shrinking. Polytetrafluoroethylene was taken as the mold material, and sculpted in the form of the corresponding female die, which was comprised of an inner core and two outer plates.

### Poration and shaping of the biological material

The biological materials exhibiting a laryngeal cartilage-like hollow, semi-flared morphology were prepared by solvent casting, compression molding and particulate filter leaching methods (8). In brief, quantitative PHBHH flocculent material (molecular weight, 600,000; provided by the Department of Chemical Engineering, Tsinghua University, Beijing, China) was placed inside a spherical, heat-resistant glass container, and chloroform solvent (Sinopharm Chemical Reagent Co., Ltd., Shanghai, China) was added (at a ratio of 2:3). The mixture was then heated for reflux under sealed conditions, stirred with a magnetic stirrer and rapidly poured into a wide-neck flask containing sodium salt (Sinopharm Chemical Reagent Co., Ltd.) (screening of 150–200 μm). Once the solution formed a uniform thin paste, the bottle was sealed and placed at room temperature overnight (making the material naturally penetrate the salt layer). The mixture of the material and the salt was firstly prepared into a sheet, then wrapped around the mold core portion, closed around the outer mold and fixed by metal fixture; the liquid was then compressed into the mold. The samples were placed in the fume cupboard for ≥48 h for full evaporation of the chloroform and subsequently placed in a vacuum pump suction filter for 12 h to remove any possible residual chloroform. The obtained samples were set within an inner metal mesh, suspended in glass containers containing distilled water, magnetically stirred and filtered for desalination. The triple-distilled water was replaced at least three times. The desalinated samples were dried naturally for porosimetry.

### Determination of PHBHH porosity

The liquid displacement method (9) was used in the determination of PHBHH porosity, Due to the lipophilic nature of the PHBHH, ethanol (Sinopharm Chemical Reagent Co., Ltd.) was used instead of water. This facilitated the penetration of the liquid into the material. The ethanol was added with a volume scale (V1) into a sealable test tube and the test sample (mass, M) was weighed and then also added to the tube. The tubes were subsequently sealed, opened after 8 h (to release the gas) and closed again. After 24 h, the ethanol penetrated sufficiently into the pores within the sample, and the volume was recorded as V2. Subsequent to removing the sample saturated with ethanol, the volume of ethanol in the tube was recorded as V3. The material density was calculated using the following formula: ρ=M/(V2−V3). The porosity (%) was calculated using the formula e=(V1−V3)/(V2−V3)×100.

### Assistance for shaping and adhesion

The shaping of the hollow, semi-flared PHBHH objects was aided by the inner surface of a steel wire with a diameter of 0.4 mm, which was uninterruptedly woven into the form of two approximate rings and three longitudinal supports. Compared with the PHBHH with the hollow semi-flared shape, the larger ring fluctuated correspondingly up and down along the semiflared shape of the corners and edges of the stent, mainly helped the shaping of the edges and rear parts with less material of the PHBHH with hollow semi-flared shape. The smaller ring was nearly round, which assisted in the shaping of the small end of the hollow, semi-flared PHBHH, and the three longitudinal wire supports were located in the sides of the PHBHH shape and in the middle of the front. The steel support frame was placed tightly inside the PHBHH model with no special fixation. The hollow, semi-flared PHBHH constructions and the steel wire support frame were disinfected by immersion into 75% alcohol and then rinsed three times with PBS, prior to being dried under sterile conditions. Adhesion was promoted by immersing the PHBHH and the frame into sterile poly-L-lysine aid (relative molecular mass, 1.89 million; Sigma-Aldrich) for 1 h, prior to drying.

### Compounding of chondrocytes with PHBHH porous materials and culturing in vitro

Following the adjustment of the cell suspension to a density of 5×10^7^/ml, the chondrocytes were inoculated in the pre-dried shaping materials by culture medium containing ~30% wet culture medium, a small amount of cell suspension each time when inoculated, was scattered and multicasted to make the cells distribute as evenly as possible, the inoculated cells of the inner surface of the shaping hollow material were pipetted using a bend head. The cells were placed in a CO_2_ saturated humidity incubator (Thermo Fisher Scientific, Inc.) at 37°C and 5% CO_2_ for 0.5 h, and then removed and agitated. The cell suspension around the bottom of the material was sucked out and inoculation was performed from top to bottom twice, prior to further incubation for 1 h. Fresh culture medium was then added slowly along the sidewall so that the composite was infiltrated by 2–3 cm. The medium was changed once every 48 h. Cell growth and adhesion in the composite edge were observed under an inverted microscope (Huxing XSP-20CD; Shanghai Huxing Optical Instrument Co., Shanghai, China). The shaped material loaded with chondrocytes was used in the experimental group, while a control group was established by shaping the PHBHH without the chondrocytes. All other steps in the two groups were identical. Each 5×5-mm sheet-like chondrocyte-PHBHH composite was fixed with 2.5% glutaraldehyde, dehydrated progressively with alcohol, dried, mounted and observed using light-emitting plasma spraying with scanning electron microscopy (SEM) (S570; Hitachi, Tokyo, Japan).

### Surgical procedure

#### Experimental grouping

Twelve male New Zealand white rabbits (age, ~6 months; weight, 2.5±0.5 kg) were randomly divided into an experimental (n=9) and a control (n=3) group. This study was carried out in strict accordance with the recommendations in the Guide for the Care and Use of Laboratory Animals of the National Institutes of Health. The animal use protocol was reviewed and approved by the Institutional Animal Care and Use Committee of the General Hospital of Shenyang Military Area Command (Shenyang, China; permit no. 20060828001).

#### Design of the pedicled myofascial flap

Intravenous anesthesia was induced with 3% barbiturate. The side of the back skin of the rabbit was disinfected and shaved, and the middle portion of the sacral spine muscle and fascia was isolated from the distal side of the rabbit’s back to form the myofascial tissue flap with the pedicle for the proximal and distal sides of the flap. The filled myofascial flap with a conical hollow portion was designed according to the size of the chondrocyte-PHBHH composite. The fascial layer of the myofascial flap, similar to a conical shape, was then stabbed into the form of a net with a scalpel blade for filling in the prepared hollow part of the composite. During flap preparation, the sacral spinal fascia in the sides of the depression was freed, simply sutured, connected and placed in the recessed slots, and the fascia in the depressed trench was subjected to poration for the *in situ* implantation of the PHBHH-chondrocyte complex. Following implantation, the remaining sacral spine muscle and fascia were separated to enclose the chondrocyte-PHBHH complex. Autologous fascia tissues were used for each implanted chondrocyte-PHBHH composite.

#### Implantation in vivo

The chondrocyte-PHBHH composites were co-cultured *in vitro* for 7–10 days, until abundant extracellular matrix was observed using inverted microscopy. The composites were removed subsequent to reaching a certain degree of support (hardness). The *in situ* implantation was designed, filled and wrapped in the body according to the design of myofascial tissue, and the distal portion of the filled myofascial flap was fixed. The subcutaneous skin was sutured. Each batch of surgical procedures was performed by the same group of technicians, and the surgical procedures and technology used were consistent. The rabbits were administered an intramuscular injection of 800,000 units penicillin, and the surgery was then repeated the next day for a total of four times.

### Experimental observation and evaluation

#### Poration, shaping and cellular compatibility of PHBHH

The PHBHH composites were observed and assessed for shapes similar to the hollow, semi-flared laryngeal cartilage morphology. The composite pore continuity and chondrocyte adhesion, distribution and growth were observed using SEM.

#### Construction of tissue-engineered, hollow, semi-flared laryngeal cartilage

The animals were anesthetized and the implants were removed: Three rabbits from the experimental group and one from the control group were selected at each time-point, six, 12 and 18 weeks after surgery, respectively. The gross morphology of the tissue-engineered laryngeal cartilage was observed, and hematoxylin and eosin (HE), Masson’s trichrome and Alcian blue/periodic acid Schiff reaction (AB/PAS) staining were performed, as well as collagen type II immunohistochemical detection for the evaluation of cartilage formation.

## Results

### Fabrication of PHBHH porous models

**Figure 1:**
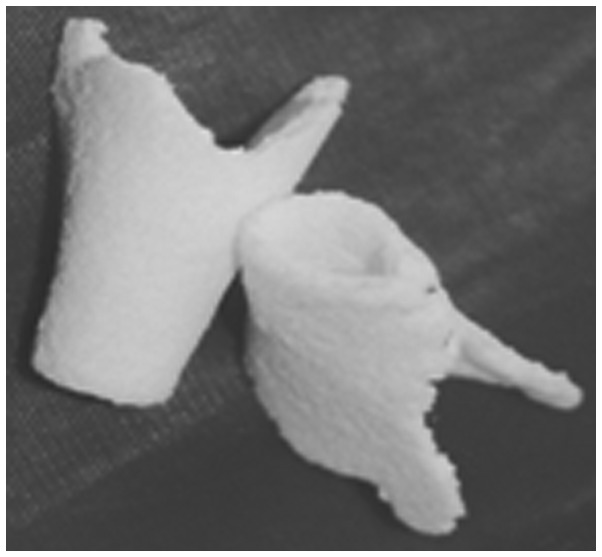
Hollow, semi-flared, porous poly(3-hydroxybutyrate-co-3-hydroxyhexanoate) biomaterials.

The porous PHBHH composite was prepared by solvent casting, compression molding and particulate filtering, and had a hollow, semi-flared shape, which was substantially similar to laryngeal cartilage morphology (Fig. 1). Following the filtering and demineralization, the whole structure was porous and spongy. The porosity was measured using the ethanol static volumetric measurement method (92±2%), and the pore size and thickness were found to be 100–150 μm and ~1.5 mm, respectively.

### Experimental observation in vitro

**Figure 2:**
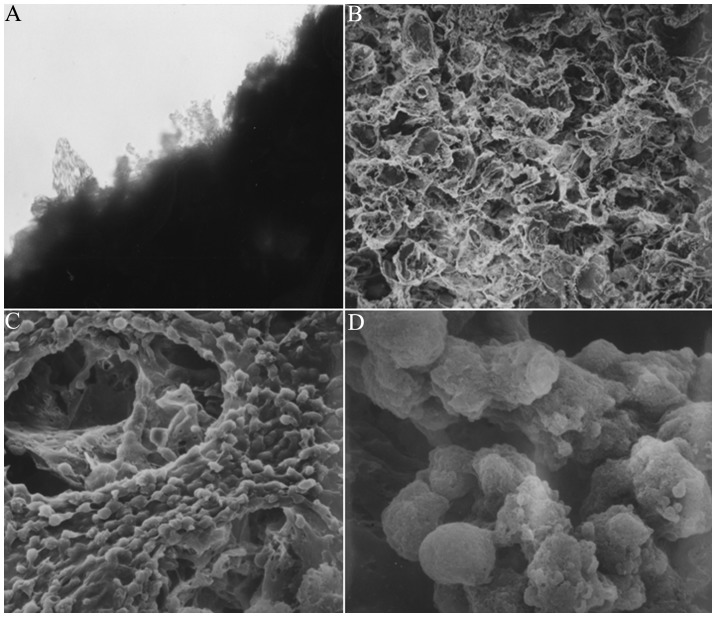
Chondrocyte-PHBHH composites cultured *in vitro* for one week. (A) Inverted microscopy showed that, at the lower edge of the composite, the cartilage cells secreted a jelly-like matrix (magnification, ×100). (B) SEM results of the porous PHBHH biomaterial (magnification, ×200). (C and D) SEM results of the chondrocyte-PHBHH composites cultured *in vitro* (magnification, ×500 and ×2,000, respectively). PHBHH, poly(3-hydroxybutyrate-co-3-hydroxyhexanoate); SEM, scanning electron microscopy.

At 24 h after the inoculation of chondrocytes into the hollow, semi-flared PHBHH biomaterials, the chondrocytes were observed to attach to the surface of the lower edge of the material under an inverted microscope. Following co-culture of the chondrocytes and PHBHH for one week, a jelly-like matrix, secreted by the chondrocytes, was visible at edge of the material (Fig. 2A).

### SEM

The simple PHBHH material was porous and spongy, exhibiting a pore size of 100–150 μm (Fig. 2B). SEM showed that the cells (single, string or cluster) were distributed at the surface and in the cavernous, spongy hollow of the chondrocyte-PHBHH complex. Mucus-like stromal substances around the cells exhibited interlinking adhesions (Fig. 2C), and numerous small projections were visible under high-magnification microscopy, which may have been fused matrix components secreted by the chondrocytes (Fig. 2D).

### General morphology

**Figure 3:**
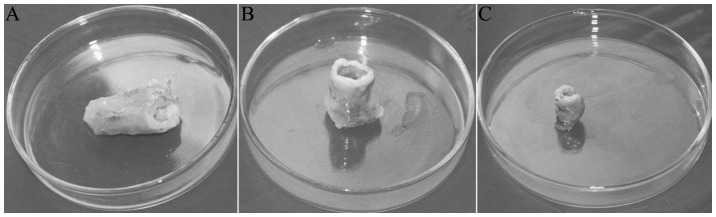
Gross specimens of the chondrocyte-PHBHH composites six weeks after implantation. (A) Gross specimen of the hollow, semi-flared, tissue-engineered laryngeal cartilage. (B) Gross specimen of the tissue-engineered laryngeal cartilage exhibiting collapse and angular loss. (C) Gross specimen of the control group (simple, hollow, semi-flared PHBHH structures placed at the corresponding time-point). PHBHH, poly(3-hydroxybutyrate-co-3-hydroxyhexanoate).

**Figure 4:**
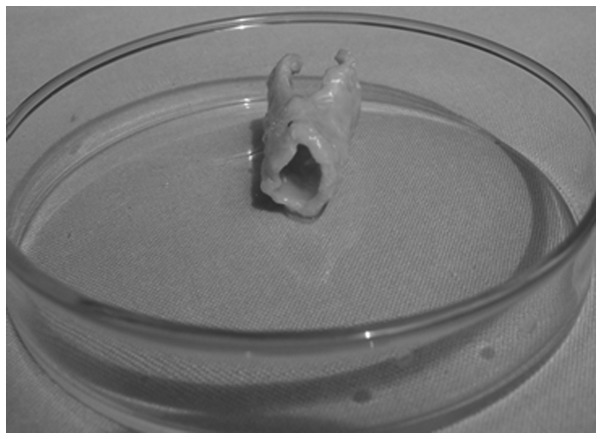
Gross specimen of the chondrocyte-poly(3-hydroxybutyrate-co-3-hydroxyhexanoate) composite 18 weeks after implantation.

The materials were harvested six weeks after implantation, and the implants and connective tissue myofascial adhesions were wrapped together. Dense, tiny blood vessels were distributed on the connective tissue. Subsequent to stripping away part of the wrapped organization it was observed that the filled myofascial tissue was closely in contact with the implants. The steel wire support frame, filled packages and fascia tissues were removed, and it was found that the general form of the implants in the two cases was consistent with the morphology prior to implantation (Fig. 3A). The specimens possessed a certain hardness with smooth inner and outer surfaces. In one case the implant had collapsed, showing angular release and a loss of the predetermined hollow, semi-flared shape (Fig. 3B). At 12 weeks, only two complete specimens of the three experimental group implants remained for harvesting, as the third collapsed; the general form showed a hollow, semi-flared, tissue-engineered laryngeal cartilage. When harvested at 18 weeks, the implant shape of the three cases was consistent, and the milky white, hollow, semi-flared, tissue-engineered laryngeal cartilage exhibited a realistic shape (Fig. 4). In the control group, one implant was removed at each corresponding time-point. The retained hollow, semi-flared shape of the gross specimens in the control group at six weeks (Fig. 3C) was significantly less than that of the experimental group at the same time-point (Fig. 3A). At 12 and 18 weeks the gross morphology of the implants in the control group had almost disappeared; only the built-in medical steel support frame was visible in the implant area.

### Histological observation

#### HE staining

**Figure 5:**
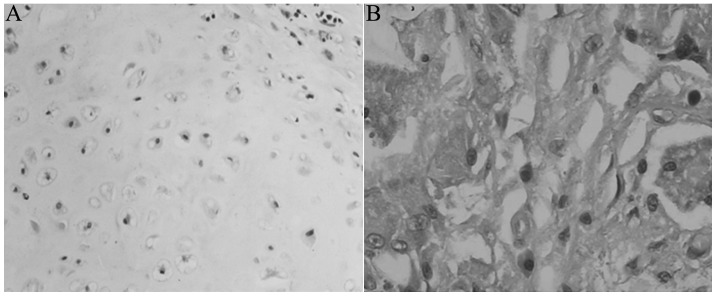
Histological observation six weeks after implantation. (A) Tissue-engineered cartilage (experimental group; HE staining; magnfication, ×200). (B) Control group [simple, hollow, semi-flared poly(3-hydroxybutyrate-co-3-hydroxyhexanoate) structures placed at the corresponding time-point] (HE staining; magnification, ×400). HE, hematoxylin and eosin.

At the six-week time-point the cells of the experimental group exhibited a circular, oval or polygonal morphology, and single cells were scattered. Incomplete degradation ‘impurities’, such as material around the PHBHH, were visible among the cells, and inflammatory cell infiltration was present around the cartilage (Fig. 5A). At 12 weeks, the majority of the cells exhibited an oval morphology; two to three cells were commonly clustered together and showed a uniform stromal staining. The morphology of the cells at 18 weeks was similar to that at 12 weeks; the ‘impurities’ had disappeared, but a small number of inflammatory cells remained in the surroundings. Cartilage-like cells in the control group were visible amongst the collapse of the pores and degradation of the material at six weeks, and few other tissue cells were scattered on the surface (Fig. 5B).

#### Masson’s trichrome staining

**Figure 6:**
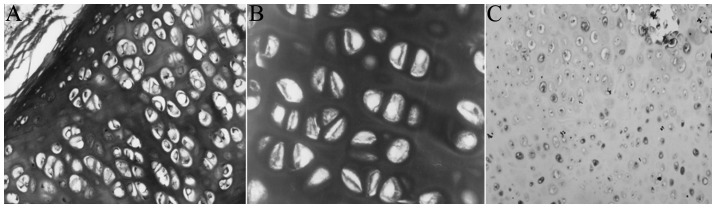
Staining and immunohistochemistry of the tissue-engineered cartilage at 12 weeks. (A) Masson’s trichrome staining (magnification, ×200). (B) Alcian blue/periodic acid Schiff reaction staining (magnification, ×400). (C) Type II collagen immunohistochemistry with 3,3′-diaminobenzidine chromogen (magnification, ×200).

Pale green objects were observed in the chondrocytes and cartilage matrix following cytoplasmic staining of the experimental group at the sixth week, and the presence of the green pollutants increased significantly at the 12th week, with deeper staining (Fig. 6A). The cell morphology, distribution and coloring at 18 weeks were not different from the observations at 12 weeks.

#### AB/PAS

The matrix was purple at the six-, 12- and 18-week time-points (Fig. 6B).

#### Immunohistochemistry for type II collagen

At six weeks, immunohistochemistry for type II collagen in the experimental group (Boster Biotechnology Co., Ltd., Wuhan, China) revealed weakly positive staining, which was mainly distributed in the cytoplasm, with very small amounts in the matrix. At 12 weeks, the positive staining in the cytoplasm was significantly enhanced, and increased type II collagen-positive staining was detected in the matrix (Fig. 6C). Similar results were observed at 18 weeks.

## Discussion

Different from the regeneration of sheet cartilage tissue by tissue engineering technology, the construction of hollow, semi-flared, tissue-engineered laryngeal cartilage has a higher demand for biocompatibility, biodegradability, pore structure, mechanical strength and the shaping of extracellular matrix materials (10). Biomaterials that are currently used in cartilage tissue engineering include natural biomaterials, such as collagen, fibrin and chitosan; synthetic biomaterials, such as polyglycolic acid; microbial biosynthetic material, such as types of biological polyhydroxyalkanoates and copolymers; and composite materials, such as polylactic acid-collagen and fibrin-polyurethane (11–14). The biomaterials each have individual advantages and disadvantages in terms of biocompatibility, degradation suitability, cell adhesion, metabolic microenvironment, mechanical strength, shaping and flexibility. Natural biological materials lack sufficient mechanical strength, while artificial and compound biomaterials are expensive and involve complex processing, but are easy to shape (15–19).

Polyhydroxyalkanoate is a natural substance produced by microorganisms and exhibits characteristics such as good biocompatibility, no induction of inflammation, no degradation and reduced rejection (20,21). As more types of polymers become available, and as the compatibility among the polymers is enhanced, tissue engineering scaffolds with improved strength, toughness, biocompatibility and controlled degradation are likely to be able to be obtained through the advantages resulting from the combination of different polymers (6).

In the present study, the selected material PHBHH, a copolymer of poly-3-hydroxybutyrate and poly-3-hydroxyhexanoate ester, not only had the advantages common to biological materials belonging to the polyhydroxyalkanoate class, but could also be found at low prices and from abundant sources. PHBHH was made into a hollow, semi-flared, tissue-engineered, larynx-shape model by solvent casting and compression molding methods; the material maintained a good shape subsequent to desalting by filtering and exhibited considerable mechanical strength. The material was porous and spongy under the electron microscope, the pore size was consistent with the screening of the salt, the porosity was high, which was in line with the basic requirements for a porous structure, and the model showed a certain degree of mechanical strength. Following the co-culture of the cartilage cells with the PHBHH composite material *in vitro*, the model showed good cell adhesion, distribution and vigorous growth; however, the mechanical strength was still insufficient to maintain the predetermined shape upon implantation, which highlighted the necessity of the auxiliary, in-built support frame. The implantation of simple PHBHH laryngeal cartilage scaffolds, without cells, was taken as the control group; after six weeks an increased loss of general shape was observed in the control group compared with the experimental group, in which a chondrocyte-PHBHH composite had been implanted. Under the microscope, no cartilage-like structure was observed in the control group, and more incomplete degradation impurities of PHBHH were apparent in the cartilage tissue. The shape of the control group implants was lost at the 12th and 18th weeks, suggesting that implantation of the material for six weeks resulted in significant, but not complete, degradation and absorption. Implantation of PHBHH alone, without seed cells, led to an ineffective generation of cartilage tissue. By contrast, after 12 weeks of *in vivo* implantation in the experimental group, mature, hollow, semi-flared, tissue-engineered laryngeal cartilage could be obtained.

Although basic research in cartilage tissue engineering has achieved encouraging results (22,23), the functional repair and reconstruction of tissue-engineered laryngeal cartilage has not yet been implemented in a practical sense (24,25). The factors impeding this implementation are the blood supply and mucosal and supportive defects of the regenerated cartilage. The limitations in the regeneration of cartilage could be overcome by combining the advantages of tissue engineering technology and the predetermination of cartilage morphology with the advantages of the use of a tissue flap to provide a blood supply. Through this strategy, and using the characteristics of epithelization (26), an affiliation could be achieved between the fibrous tissues and the tissue flap. The tissue flap could be prepared using the design principle of filling the lining of the cavity fascia and wrapping the outer cavity with muscle to adhere to the tissue-engineered cartilage. The complex could then be used to reconstruct the laryngeal cartilage with scaffold functions according to the common nearest transfer or graft method (27–31). This technique is likely to overcome the problems in vascularization, mucosalization and support in cartilage repair and reconstruction for laryngeal cartilage.

Based on the above idea, the present study attempted to take a fascia tissue lining and use myofascial tissue flap filling and wrapping technology to build a predetermined, hollow, semi-flared, tissue-engineered laryngeal cartilage. In the experimental procedure, the myofascial tissue of the fascial portion was placed on the surface of the proposed cartilage construction for contact. Fascia poration not only enhanced blood circulation, but was also conducive to adhesion between the structures. Experimentally designed muscle flap was filled with a rich blood supply, while the seperated flap, which remained in the recessed part, also formed an equally rich blood supply. The cultured chondrocyte-hollow, semi-flared PHBHH composite was placed off the depression of the tissue flap and cultured for a certain period of *in vitro*. The cavity was filled with fascia and muscle lining, and the periphery was supported by the tissue around depression or recessed slots. The upper part of the complex was covered and wrapped with myofascial tissues. The results of the study showed that the application of myofascial tissue with a flap blood supply with functional filling and wrapping technology could successfully lead to the generation of hollow, semi-flared, tissue-engineered laryngeal cartilage. Difficulties may arise, however, in avoiding the collapse of the samples, considering the multiple steps in the construction of the hollow, semi-flared, tissue-engineered laryngeal cartilage.

This study has raised the potential for a common transfer transplantation with complex of the tissue flap and hollow, semi-flared, tissue-engineered cartilage, and has indicated the success of the myofascial flap filling and wrapping method in generating hollow, semi-flared, tissue-engineered laryngeal cartilage for functional repair and reconstruction. The study has also demonstrated the potential of the mucosal epithelial transformation of an ideal hollow cartilage surface; however, the results of this study are preliminary, and the design of laryngeal cartilage scaffolds based on this design concept, and the construction, transplantation and practical applications of the composite tissues, should be the direction of future studies.
